# Stochastic optimization of a uranium oxide reaction mechanism using plasma flow reactor measurements

**DOI:** 10.1038/s41598-023-35355-6

**Published:** 2023-06-07

**Authors:** Mikhail Finko, Batikan Koroglu, Kate E. Rodriguez, Timothy P. Rose, Jonathan C. Crowhurst, Davide Curreli, Harry B. Radousky, Kim B. Knight

**Affiliations:** 1 Lawrence Livermore National Laboratory, Livermore, CA 94550 USA; 2 Department of Nuclear, Plasma, and Radiological Engineering, University of Illinois Urbana-Champaign, Champaign, IL 61820 USA

**Keywords:** Reaction mechanisms, Computational science, Optical spectroscopy, Analytical chemistry

## Abstract

In this work, a coupled Monte Carlo Genetic Algorithm (MCGA) approach is used to optimize a gas phase uranium oxide reaction mechanism based on plasma flow reactor (PFR) measurements. The PFR produces a steady Ar plasma containing U, O, H, and N species with high temperature regions (3000–5000 K) relevant to observing UO formation via optical emission spectroscopy. A global kinetic treatment is used to model the chemical evolution in the PFR and to produce synthetic emission signals for direct comparison with experiments. The parameter space of a uranium oxide reaction mechanism is then explored via Monte Carlo sampling using objective functions to quantify the model-experiment agreement. The Monte Carlo results are subsequently refined using a genetic algorithm to obtain an experimentally corroborated set of reaction pathways and rate coefficients. Out of 12 reaction channels targeted for optimization, four channels are found to be well constrained across all optimization runs while another three channels are constrained in select cases. The optimized channels highlight the importance of the OH radical in oxidizing uranium in the PFR. This study comprises a first step toward producing a comprehensive experimentally validated reaction mechanism for gas phase uranium molecular species formation.

## Introduction

The reaction kinetics of gas phase metal oxides are of broad relevance to many research fields, including astrophysics, combustion science, nuclear engineering, and material chemistry in extreme environments. In recent years, the latter field has produced numerous experimental and computational works on uranium oxide (${{\mathrm{UO_x}}}$) vapor chemistry^1^. Gas phase products of refractory oxides, like ${{\mathrm{UO_x}}}$, have historically been challenging to produce due to the high vaporization temperatures of the parent oxides. More recently, thermal plasma systems have provided an avenue for readily producing gas phase metals and studying their chemistry in reactive environments. However, the rapid quenching times, presence of background radicals, and formation of volatile intermediate oxides in such systems make it difficult to isolate specific reaction channels for study. Similar problems arise in other reactive high temperature systems like metal combustion fuels. As a result, gas phase metal oxidation mechanisms are often based on sparse experimental data and first-order theoretical estimates, as for aluminum oxide formation^2–4^. Likewise, a ${{\mathrm{UO_x}}}$ reaction mechanism was constructed using a comparable methodology in our previous work^5^. Although such mechanisms produce qualitatively reasonable results that may align with some experimental observables, detailed experimental validation is difficult to achieve. This validation step is crucial for ensuring that the chemical kinetic mechanism can be used in a predictive manner to inform subsequent models. Here, we explore a method of inferring rate coefficients of uranium oxide (${{\mathrm{UO_x}}}$) based on experimental measurements from a thermal plasma system.

Due to the strongly coupled and non-linear nature of chemical kinetics in uranium plasmas, extracting reaction rate information necessitates solving an optimization problem. In this problem, the underlying model parameters (rate coefficients) are determined based on observed outputs (i.e. spectroscopic information). Solving such a problem by deterministic gradient-based methods is difficult due to the potentially complex parameter space with numerous local minima. In this case, one must instead utilize an optimization method capable of continuously exploring the entire parameter space while locating the global minimum. One such method previously used for chemical kinetics problems is the Monte Carlo genetic algorithm (MCGA)^6^. This technique is well suited for the current problem due to its effectiveness in avoiding convergence on local minima and its ease of implementation. Regardless of methodology, solving an optimization problem requires repeated evaluations of the associated model, often numbering in the thousands to millions of runs. While reasonable computational times are achieved when solving for the chemical kinetics in a spatially uniform system, the problem quickly becomes unfeasible when the chemistry is coupled with complex fluid transport. This consideration becomes important when choosing an experimental system for informing the optimization problem.

Experimental systems suitable for informing the uranium-oxygen reaction mechanism optimization at atmospheric pressure include laser ablation systems^7–14^ and a plasma flow reactor (PFR)^15,16^. Laser ablation systems utilize a high-intensity pulsed laser to volatilize metal samples, producing rapidly expanding reactive plasma plumes. If performed by a sufficiently powerful laser in atmospheric conditions, the ablation is accompanied by a shock wave at the plume-ambient interface reminiscent of a fireball blast wave. The chemical composition of the plume as a function of time can then be measured using optical spectroscopy. The plasma flow reactor, on the other hand, produces a uranium plasma using an inductively coupled plasma (ICP) torch attached to a quartz tube. While the RF plasma is generated by an argon flow, an aqueous uranium solution is introduced into the torch, producing a uranium bearing plasma that cools as it flows downstream. Optical spectroscopy is used to measure the chemical evolution of select species in the plasma at various points along the tube.

Although both experimental systems could theoretically be used for optimizing a reaction mechanism, a major advantage of using the plasma flow reactor is that the species residence time can be correlated with the distance along the reactor. That is, if we follow a parcel of fluid with a known starting chemical composition through the reactor, its chemical composition at a given position can be related to its residence time via the flow rate. This Lagrangian approach allows the chemical evolution in a plasma flow reactor to be approximated using a purely transient model, such as a global kinetic model^15^. In contrast, the complex transport behavior of a laser ablation plume requires employing a more computationally expensive model, such as a reactive compressible fluid model^17^. Based on our previous experience with the above simulations, a global kinetic model of the flow reactor completes in a matter of seconds, whereas a fluid laser ablation model might take hours or longer to complete. The significant reduction in computational effort required to model the plasma flow reactor is critical for optimization and motivates the use of the system in this work. Below, we outline how the PFR experiments, modeling, and MCGA optimization are carried out to produce an experimentally constrained ${{\mathrm{UO_x}}}$ reaction mechanism.

## Methodology

### System description

**Figure 1:**
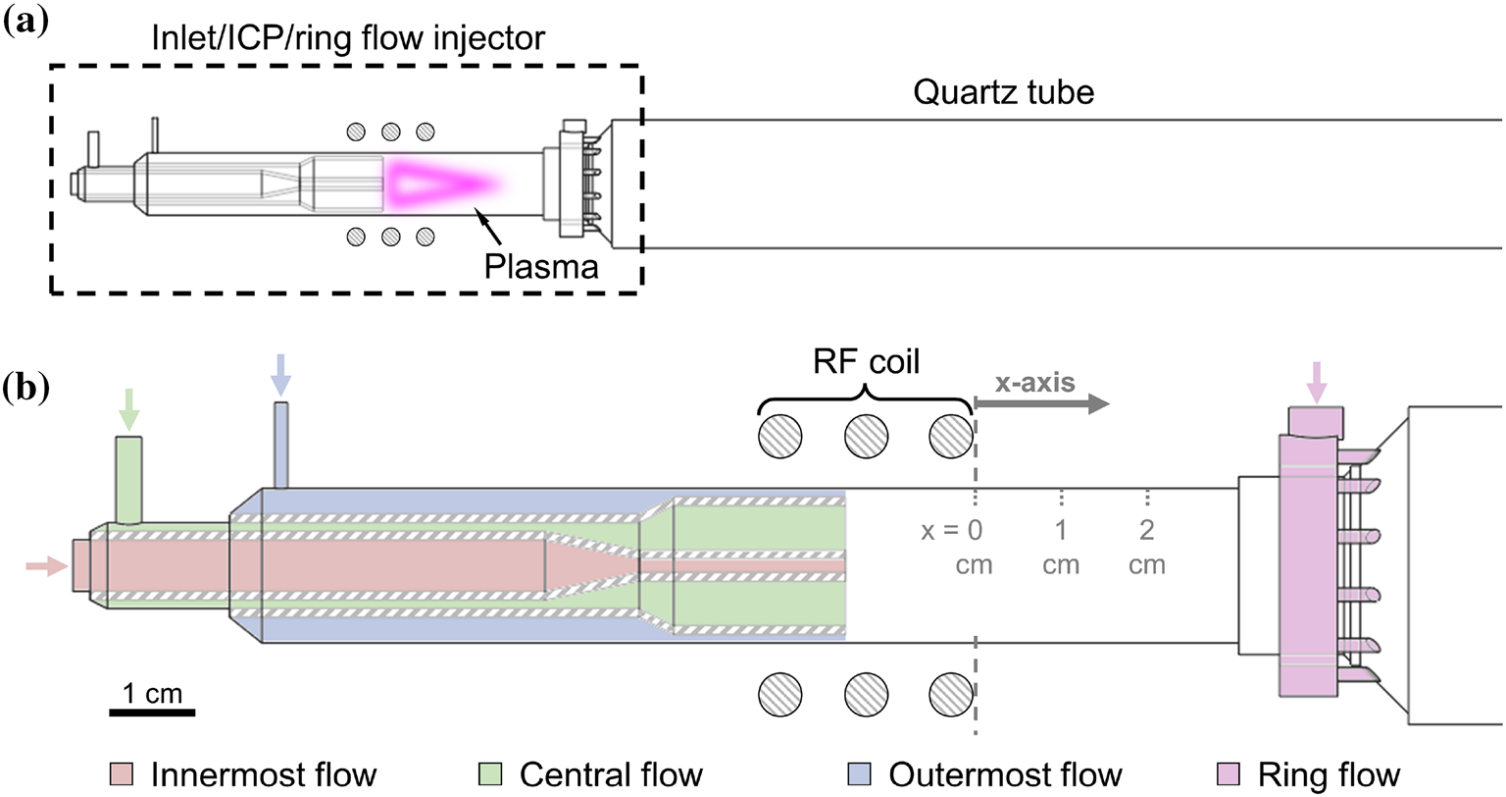
Diagram of the plasma flow reactor. Sub-figure (**a**) shows the upstream nozzle injector region, plasma location, and downstream quartz tube. Sub-figure (**b**) highlights the three concentric annular inlet flow channels, the location of the inductively coupled plasma (ICP) coil, and the optional ring flow injector of the inlet region.

The PFR is a commercially available ICP system that was modified^18^ to study gas phase chemical kinetics and nanoparticle formation and growth. A diagram of the PFR used in this work is shown in Fig. 1. The inlet region of the PFR consists of three concentric annular flow channels, each with a separate flow rate and composition. An aqueous uranyl nitrate solution (${{\textrm{UO}}}_2({{\mathrm{NO_3}}})_2\cdot {{\mathrm{6H_2O}}}$) is nebulized into liquid droplets and introduced via the innermost channel (marked in red) using a carrier gas (argon). For a typical argon gas flow rate of 1 L/min, the uranium is about four orders of magnitude less abundant than argon in the innermost flow (i.e. $\sim$100 ppm). To enhance oxidation kinetics, oxygen gas can be added through this channel, with typical flow rates of 10–50 mL/min. In addition, the outermost channel (marked in blue) provides an added 12–14.4 L/min of argon gas flow to sustain the plasma and to cool the outer quartz wall. The central channel (marked in green) is not used in these experiments. Based on the above flow rates, analyte concentrations are on the order of 10 to 100 ppm in the downstream flow, depending on the extent of radial mixing and diffusion. The number densities, flow rates, and composition of the fluid components prior to entering the plasma are listed in Table 1, with uranium nitrate split into its component molecules for convenience.

**Table 1:** Number densities, flow rates, and composition of the inlet flow components in the PFR.

	Analyte			Added oxygen	Carrier gas
	$\hbox {UO}_2$	$\hbox {H}_2$O	$\hbox {NO}_3$	$\hbox {O}_2$	Ar
*n* (cm$^{-3}$)	$3.48\times 10^{22}$			$2.45\times 10^{19}$	$2.45\times 10^{19}$
${\dot{V}}$ (L/min)	$2.52\times 10^{-5}$			0–0.05	1–15.4
Species fraction	$4.31\times 10^{-3}$	$9.87\times 10^{-1}$	$8.62\times 10^{-3}$	1	1
${\dot{N}}$ (molecules/min)	$3.78\times 10^{18}$	$8.65\times 10^{20}$	$7.56\times 10^{18}$	0–$1.22 \times 10^{21}$	(0.25–$3.77) \times 10^{23}$

A 40 MHz RF plasma is generated downstream of the inlet channels using an inductive coil surrounding the outer quartz tube. Since the majority of the inlet flow is argon and the plasma is generated at atmospheric pressure, the thermodynamic and transport properties of the plasma can be closely approximated as that of an LTE argon plasma^19,20^. The plasma and downstream flow temperatures can be modified by adjusting the outermost argon flow rate and the power provided by the power supply. Lastly, an optional ring flow injector can be used to introduce additional argon flow further downstream of the RF coil, although this feature was not utilized in this work. Alternatively, a constant diameter quartz tube extension can be connected to the torch when the ring flow injector is not needed.

Optical emission spectroscopy (OES) is used to track the evolution of U and UO in the PFR. Light emitted by the plasma is routed to a spectrometer using a fiber optic cable positioned at various axial locations along the flow reactor. A motorized linear translation stage is used to move the fiber optic cable along the x-axis denoted in Fig. 1, keeping the fiber at a fixed radial distance away from the reactor center. The end of the RF coil is used as the reference $x=0$ axial location for all measurements (as shown in Fig. 1).

Both the ring flow and constant diameter configurations described above feature optically opaque regions where the flow emission is obscured. These regions cover 0–3 cm and 3–5 cm from the RF coil for the constant diameter extension and the ring flow injector, respectively. Furthermore, as the fiber optic tip is conductive and was not insulated, the minimum axial distance from the RF coil was kept to 1 cm to prevent arcing. Since the flow characteristics in the torch region should be identical for both configurations, the observation limitations are overcome by combining upstream and downstream data taken with the ring flow and constant diameter configurations, respectively.

### Monte Carlo genetic algorithm

Calibrating a reaction mechanism with respect to experimentally measured quantities is an example of an inverse problem, that is, one where the governing equations and solution are known, but the input parameters are not. This type of problem typically does not admit a unique solution and is instead posed as an optimization problem where the suitability of a solution is dictated by an objective function. The objective function quantifies the statistical deviation of the calculated solution from the true solution. For example, a common objective function is the sum of the squares of the solution residuals:

1 $$\begin{aligned} \phi ({\varvec{k}}) = \sum _i^{\textrm{time}} \left[ {\varvec{n}}^{exp}_i - {\varvec{n}}^{calc}_i({\varvec{k}})\right] ^2 \end{aligned}$$

where ${\varvec{k}}$ is a vector containing the reaction rate coefficients and $n^{exp}_i$ and $n^{calc}_i({\varvec{k}})$ are the measured and calculated species number densities at time point *i*, respectively. The optimization problem is solved by employing an iterative procedure that finds an optimal parameter set ${\varvec{k}}$ that minimizes the objective function $\phi$. In the context of the current problem, an optimized ${\varvec{k}}$ value would represent a set of rate coefficients that closely match the uranium oxide formation rates observed in the laser ablation or PFR experiments. Typically, deterministic nonlinear least squares methods, such as the Gauss-Newton or Levenberg-Marquadt methods^21–23^, are employed for such optimization problems. Modern computational techniques, such as neural networks^24^, can also be used to this end.

Due to the large parameter space of the ${{\mathrm{UO_x}}}$ reaction mechanism optimization problem, the solution space may be complex and may contain numerous local minima. Conventional deterministic optimization methods struggle with locating a global minimum for such a problem, instead converging to local minima adjacent to the initialization point. Exhaustive search methods are similarly ineffective due to the computational demand of mapping out a large parameter space. To avoid these issues, we employ a Monte Carlo Genetic Algorithm (MCGA) approach^6^ to optimize the ${{\mathrm{UO_x}}}$ reaction mechanism. This approach combines the Monte Carlo and genetic algorithm stochastic optimization methods to achieve global optimization for problems with large parameter spaces. The Monte Carlo portion of the approach uses random sampling of reaction rate parameters to locate regions of good fitness within the solution space. The genetic algorithm then optimizes these regions to find the global minimum by using evolutionary processes of migration, selection, mating, and mutation. The stochastic nature of these processes maintains diversity among the optimized parameter sets, thereby avoiding convergence to local minima. Thus, the MCGA approach enables global optimization by searching the entire solution space using Monte Carlo sampling coupled with the parametric diversity inherent in genetic algorithm optimization. Furthermore, MCGA can be easily adapted to different experimental systems, since the governing equations of the modeled system need not be reformulated as an inverse problem. Lastly, MCGA is easy to parallelize: the objective function for each parameter set can be evaluated independently, so the evaluation process can be freely split between processors. The robustness, flexibility, and speed of the MCGA approach makes it an excellent tool for producing an experimentally calibrated reaction mechanism for uranium oxide formation.

**Figure 2:**
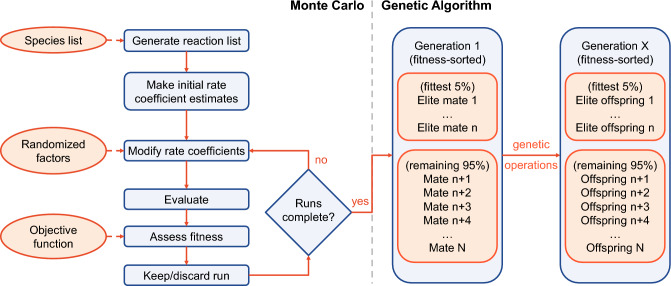
Diagram of the Monte Carlo (left of dashed line) and genetic algorithm (right of dashed line) portions of the Monte Carlo Genetic Algorithm (MCGA). The set of reaction mechanisms produced by the Monte Carlo process serves as the starting population for the genetic algorithm, where each mate is a reaction mechanism. The genetic operations responsible for producing subsequent generations are detailed further below in Fig. 4.

A diagram outlining the MCGA approach is shown in Fig. 2. Following the diagram, the Monte Carlo process can be separated into several key tasks: reaction mechanism generation, rate coefficient modification, model evaluation, and fit assessment. Each of these tasks, and how they fit into the Monte Carlo portion of the algorithm, are detailed below in correspondingly named subsections. All but the first of these tasks are also present in the genetic algorithm, as discussed in the last subsection here.

### Reaction mechanism generation

The first step of the MCGA process is to specify the set of reaction channels (a reaction mechanism) that will be used for evaluating the chemical behavior of the system. The reaction mechanism consists of two parts: a set of uranium reaction channels that are the target of the optimization and a set of supporting reaction channels responsible for the background chemistry. Due to the uranyl nitrate solution used in the PFR, the latter mechanism consists of various gas-phase^25–27^ and plasma-phase^25,28–30^ reaction channels that detail the chemical behavior of an O-H-N plasma. To reduce the computational cost of evaluating the background chemistry, a reaction mechanism reduction step is performed on this O-H-N mechanism. The reduction is performed by excluding molecules for which formation is unfavorable in the conditions of interest (2000–5000 K), as well as eliminating extensive reaction networks that track excited atomic and molecular states of minor species. For example, since nitrogen is present in small quantities, reactions involving it are reduced considerably, minimally affecting the calculation results while reducing computational time appreciably. Each step of the reduction was checked by running a test 0D simulation and verifying that formation of ${{\mathrm{UO_x}}}$ species was minimally affected. Furthermore, the reduced mechanism was verified after each MCGA optimization by testing the resulting U-O mechanisms with both the reduced and full O-H-N mechanisms, finding good agreement. The final reduced O-H-N mechanism consists of 44 species and 166 reaction channels, compared to 81 species and 796 reaction channels for the full mechanism.

**Table 2:** U-O reaction channels targeted for optimization and initial estimated and minimum modified rate coefficients.

No.	Reaction	$\Delta _r H_{298.15K}$ (kJ/mol)	$k_{est}$			$k_{mod,min}$		
			*A* (cm$^3$/s)	*n* (–)	$E_A/R$ (K)	*A* (cm$^3$/s)	*n* (–)	$E_A/R$ (K)
1	${\mathrm{U + O}} \rightleftharpoons {\textrm{UO}}$	− 758.237	$2.093 \times 10^{-11}$	0.5	0.0	$2.093 \times 10^{-15}$	− 2.5	$10^{4.6}$
2	${\mathrm{U + O_2}} \rightleftharpoons {\mathrm{UO_2}}$	− 1011.363	$1.707 \times 10^{-11}$	0.5	0.0	$1.707 \times 10^{-15}$	− 2.5	$10^{4.6}$
3	${\mathrm{U + O_2}} \rightleftharpoons {\mathrm{UO + O}}$	− 259.889	$1.707 \times 10^{-11}$	0.5	0.0	$1.707 \times 10^{-15}$	− 2.5	$10^{4.6}$
4	${\mathrm{U + OH}} \rightleftharpoons {\mathrm{UO + H}}$	− 328.366	$2.114 \times 10^{-11}$	0.5	0.0	$2.114 \times 10^{-15}$	− 2.5	$10^{4.6}$
5	${\mathrm{U + H_2O}} \rightleftharpoons {\mathrm{UO + H_2}}$	− 267.238	$2.130 \times 10^{-11}$	0.5	0.0	$2.130 \times 10^{-15}$	− 2.5	$10^{4.6}$
6	${\mathrm{UO + O}} \rightleftharpoons {\mathrm{UO_2}}$	− 751.474	$2.116 \times 10^{-11}$	0.5	0.0	$2.116 \times 10^{-15}$	− 2.5	$10^{4.6}$
7	${\mathrm{UO + O_2}} \rightleftharpoons {\mathrm{UO_3}}$	− 823.213	$1.722 \times 10^{-11}$	0.5	0.0	$1.722 \times 10^{-15}$	− 2.5	$10^{4.6}$
8	${\mathrm{UO + O_2}} \rightleftharpoons {\mathrm{UO_2 + O}}$	− 253.126	$1.722 \times 10^{-11}$	0.5	0.0	$1.722 \times 10^{-15}$	− 2.5	$10^{4.6}$
9	${\mathrm{UO + OH}} \rightleftharpoons {\mathrm{UO_2 + H}}$	− 321.603	$2.136 \times 10^{-11}$	0.5	0.0	$2.136 \times 10^{-15}$	− 2.5	$10^{4.6}$
10	${\mathrm{UO + H_2O}} \rightleftharpoons {\mathrm{UO_2 + H_2}}$	− 260.475	$2.152 \times 10^{-11}$	0.5	0.0	$2.152 \times 10^{-15}$	− 2.5	$10^{4.6}$
11	${\mathrm{U + O}} \rightarrow {\mathrm{UO^+ + e^-}}$	− 201.098	$2.093 \times 10^{-11}$	0.5	0.0	$2.093 \times 10^{-15}$	− 2.5	$10^{4.6}$
12	${\mathrm{U + O_2}} \rightarrow {\mathrm{UO_2^+ + e^-}}$	− 475.259	$1.707 \times 10^{-11}$	0.5	0.0	$1.707 \times 10^{-15}$	− 2.5	$10^{4.6}$

The uranium reaction channels targeted for optimization are listed in Table 2. Only reactions that are constrained by available experimental data and system conditions are included to avoid possible over-fitting from poorly constrained reactions. For example, since the reactive species are dilute in the flow, three-body reactions with a non-Ar third body will be infrequent and can be excluded. Furthermore, since the system pressure is kept constant, reaction pressure dependence is unconstrained. Therefore, the list of possible reactions is limited to bimolecular reactions, dramatically reducing the number of potential pathways. Furthermore, since the emission measurements comprising the datasets are limited to U and UO, the chemistry of higher uranium oxides is not well constrained. While the measurements provide some constraints on the formation of $\hbox {UO}_2$ via the UO consumption rate, they contain no information regarding the $\hbox {UO}_2$ consumption and $\hbox {UO}_3$ formation rates. Therefore, only reactions involving either U or UO in the exothermic direction are considered for optimization. Note that Table 2 also includes two associative ionization reactions from our previously constructed reaction mechanism^5^. These reactions have a large impact on the uranium plasma chemistry due to the nearly hard sphere reaction rate for the ${{\mathrm{U + O}}}$ associative ionization channel^31^. However, to our knowledge, this behavior is not well validated. Therefore, we include these channels in the optimization to determine the importance of associative ionization pathways for ${{\mathrm{UO_x}}}$ formation. Although ${{\mathrm{UO^+}}}$ and ${{\mathrm{UO^+_2}}}$ are not directly measured here, these reactions are partially constrained by the available U and UO data.

The starting rate coefficients ($k_{est}$) for each reaction in Table 2 are estimated using various first order approximations^32^ expressed in a modified Arrhenius type-form:

2 $$\begin{aligned} k_{est} = AT^n\exp \left( -\frac{E_A}{RT}\right) \end{aligned}$$

where *A* is the collision frequency, *T* is the gas temperature, *n* is a temperature power constant, $E_A$ is the activation energy, and *R* is the gas constant. The Simple Collision Theory (SCT) and Simplified Model of Triple Collisions (SMTC) methods are used for calculating binary and three-body rate coefficients, respectively. The collision cross sections for molecules are estimated from the bond lengths and combined Van der Waals volumes of the constituent atoms. These estimates provide an upper bound on the collision frequency *A* and a temperature power constant $n=0.5$ due to a thermal velocity contribution. No a priori estimates are made for the activation energy $E_A$; all reaction channels are initially assumed to be barrierless. The reaction channels are expressed in the exothermic direction to avoid unphysically high reverse reaction rates. Note also that bimolecular association reactions are formulated in the high-pressure limit to provide an upper bound rate estimate as the starting point.

**Table 3:** U-O reaction channels from previously constructed reaction mechanism^5^ that are included in the 0D model but are not optimized due to a lack of constraining data.

No.	Reaction	$\Delta _r H_{298.15K}$ (kJ/mol)	*k*		
			*A* (cm$^{3(m-1)}$/s)^a^	*n* (–)	$E_A/R$ (K)
13	${\mathrm{UO_2 + O_2}} \rightleftharpoons {\mathrm{UO_3 + O}}$	− 74.129	$1.17 \times 10^{-11}$	0.5	8915.7
14	${\mathrm{UO_3}} \rightleftharpoons {\mathrm{UO_2 + O}}$	570.083	$1.00 \times 10^{+15}$	0.0	73300.3
15	${\mathrm{e^- + U}} \rightarrow {\mathrm{U^+ + 2e^-}}$	604.421	$1.000 \times 10^{-4}$	− 0.721	80587.0
16	${\mathrm{e^- + UO}} \rightarrow {\mathrm{UO^+ + 2e^-}}$	550.956	$1.715 \times 10^{-10}$	0.634	84323.0
17	${\mathrm{e^- + UO_2}} \rightarrow {\mathrm{UO_2^+ + 2e^-}}$	529.925	$9.452 \times 10^{-9}$	0.256	84862.0
18	${\mathrm{U + O_3}} \rightarrow {\mathrm{UO_2^+ + O + e^-}}$	− 367.889	$7.750 \times 10^{-13}$	0.5	0.0
19	${\mathrm{U + O_3}} \rightarrow {\mathrm{UO^+ + O_2 + e^-}}$	− 93.728	$7.750 \times 10^{-13}$	0.5	0.0
20	${\mathrm{U^+ + O_2}} \rightleftharpoons {\mathrm{UO^+ + O}}$	− 313.358	$3.978 \times 10^{-10}$	0.0	0.0
21	${\mathrm{UO^+ + O_2}} \rightleftharpoons {\mathrm{UO_2^+ + O}}$	− 274.161	$2.477 \times 10^{-10}$	0.0	0.0
22	${\mathrm{UO_2^- + O_2}} \rightleftharpoons {\mathrm{UO_3^- + O}}$	− 182.126	$1.481 \times 10^{-10}$	0.0	0.0
23	${\mathrm{UO + O^-}} \rightleftharpoons {\mathrm{UO_2^-}}$	− 699.408	$3.037 \times 10^{-9}$	0.0	0.0
			$1.379 \times 10^{-8}$	− 0.5	0.0
24	${\mathrm{UO + O_2^-}} \rightleftharpoons {\mathrm{UO_2^- + O}}$	− 300.364	$1.105 \times 10^{-9}$	0.0	0.0
			$5.016 \times 10^{-9}$	− 0.5	0.0
25	${\mathrm{UO + O_3^-}} \rightleftharpoons {\mathrm{UO_2^- + O_2}}$	− 530.513	$9.272 \times 10^{-10}$	0.0	0.0
			$4.209 \times 10^{-9}$	− 0.5	0.0
26	${\mathrm{UO_2 + O^-}} \rightleftharpoons {\mathrm{UO_3^-}}$	− 628.404	$3.056 \times 10^{-9}$	0.0	0.0
27	${\mathrm{UO_2 + O_2^-}} \rightleftharpoons {\mathrm{UO_3^- + O}}$	− 229.360	$1.110 \times 10^{-9}$	0.0	0.0
28	${\mathrm{UO_2 + O_3^-}} \rightleftharpoons {\mathrm{UO_3^- + O_2}}$	− 459.509	$9.302 \times 10^{-10}$	0.0	0.0
29^b^	${\textrm{UO}}^+_x{\mathrm{+ e^- + e^-}} \rightarrow {\textrm{UO}}_x{\mathrm{+ e^-}}$	N/A$^{{\textrm{c}}}$	$9.821 \times 10^{-9}$	− 9/2	0.0
30^b^	${\textrm{UO}}^+_x{\mathrm{+ e^- + M}} \rightarrow {\textrm{UO}}_x{\mathrm{+ M}}$	N/A$^{{\textrm{c}}}$	$3.118 \times 10^{-23}$	− 3/2	0.0

^a^Units *m* is the reaction order (i.e. 1/s, cm$^3$/s, cm$^6$/s for 1st, 2nd, 3rd order reactions).

^b^0 $$< x<$$ 2.

^c^Varies depending on the value of *x*.

In addition to the ${{\mathrm{UO_x}}}$ reaction channels subject to optimization by the MCGA, we also consider several supplementary uranium reaction pathways that are not adjusted by the algorithm. These reaction channels are shown in Table 3 and consist mainly of plasma chemical reactions (ionization, recombination, charge exchange) as well as reactions between $\hbox {UO}_2$ and $\hbox {UO}_3$. The reaction rates of these channels are kept fixed due to a lack of constraining experimental data. Nevertheless, these reactions provide pathways for uranium plasma chemistry and higher oxide formation within the model.

### Rate coefficient modification

Once the target U-O reaction mechanism is generated, the main Monte Carlo loop begins. Each iteration of this loop is independent and consists of evaluating and assessing a modified version of the generated U-O reaction mechanism. Each modified mechanism is produced by adjusting the Arrhenius parameters of the original mechanism as follows:

3 $$\begin{aligned} k_{mod} = k_{est} f T^m \exp (-e/T) \end{aligned}$$

where *f* is a factor between $10^{-4}$ and $10^0$ randomly sampled from a base 10 log uniform distribution, *m* is a factor between $-3$ and 0 randomly sampled from a uniform distribution, and *e* is a factor between $10^0$ and $10^{4.6}$ randomly sampled from a base 10 log uniform distribution. The bounds of these factors are chosen so as not to exceed the physical upper limit provided by the initial hard sphere rate estimates. Thus, the $k_{est}$ and $k_{mod,min}$ values shown in Table 2 represent the upper and lower bounds of the modified rates, respectively. The factor *f* is intended to compensate for the overestimation of the collision frequency *A* provided by the initial hard sphere rate estimate $k_{est}$. The factor *m* represents a change to the temperature dependence *n* of the modified Arrhenius form. The factor *e* represents an adjustment to the activation energy $E_A/R$. The upper bound value of $e=10^{4.6}$ is chosen so that the exponential part of Eq. (3) reduces the reaction rate by four orders of magnitude at the peak plasma temperature of $\sim 4500$ K. Therefore, if the maximum value of the activation energy is used, the reaction channel is effectively removed from the reaction mechanism, indicating that the activation energy is too high for the reaction to occur in the current system. Conversely, an activation energy of $10^0$ indicates that virtually no activation barrier is present for the reaction, and the exponential term in Eq. (3) will have little effect on the reaction rate.

### Kinetic model evaluation

The modified reaction mechanism is evaluated using a global kinetic model in order to calculate the chemical evolution inside the plasma flow reactor. The model solves for the transient chemical balance of a closed (adiabatic) spatially uniform (0D) system. The model consists of a system of strongly coupled ordinary differential equations, each governing the concentration of a particular chemical species:

4 $$\begin{aligned} \frac{\textrm{d} n_i}{\textrm{d} t} = \sum ^{\textrm{reactions}}_j {\dot{S}}_{ij} \end{aligned}$$

where $n_i$ is the number density of species *i* and ${\dot{S}}_{ij}$ is a source term describing the contribution of an elementary reaction *j* to the production or consumption of species *i*. ${\dot{S}}_{ij}$ is given by:

5 $$\begin{aligned} {\dot{S}}_{ij} = \Delta c_{ij} {\dot{R}}_j = \Delta c_{ij} k_j \left( \prod ^{\textrm{reactants}}_s \left( n_s\right) ^c\right) _j \end{aligned}$$

where $\Delta c_{ij}$ is the net stoichiometric coefficient for species *i* in reaction *j*, ${\dot{R}}_j$ is the reaction rate for reaction *j*, $k_j$ is the rate coefficient for reaction *j*, and *c* is the stoichiometric coefficient of reactant *s* in reaction *j*. If a reaction is reversible, the forward and backward reaction rates ($k_+$ and $k_-$, respectively) are determined using the principle of detailed balance via an equilibrium coefficient $K_{eq} = k_+/k_-$. The equilibrium coefficient is determined by the thermodynamic properties of the species participating in the reaction^5^.

**Figure 3:**
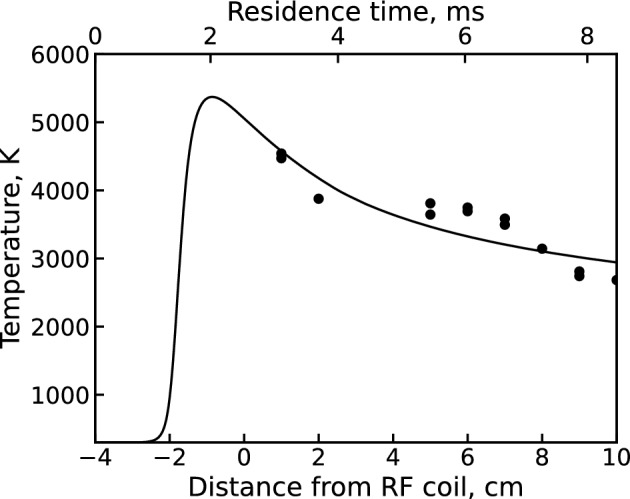
Lagrangian temperature history produced by a CFD model of the PFR^18^ (solid line) vs experimental temperature values obtained from previous Fe atomic line measurements via the Boltzmann plot method (points).

Typically, the system of species balance equations is supplemented by a heat balance equation that accounts for Ohmic heating, chemical energy release, and convective/conductive/radiative cooling in the plasma flow. Here, we instead interpolate the plasma temperature at each time step based on an experimentally calibrated temperature profile. Doing so both lowers the model’s computational complexity and improves consistency between the modeled and experimental temperature conditions. A previously developed CFD model^18^ is used to track the temperature in the PFR along a Lagrangian streamline. The Lagrangian fluid parcel trace provides both a temperature profile and a time/distance correlation for expressing the time-dependent 0D concentrations in terms of axial location. Figure 3 compares this Lagrangian temperature profile against available experimental temperature measurements. The experimental temperatures are extracted from the relative intensities of atomic Fe lines (using an iron nitrate analyte) and the corresponding line transition probabilities using the Boltzmann plot method^18^. Note that this method of temperature determination was previously found to yield consistent values within measurement uncertainty for iron, aluminum, and cerium nitrates^15,33^, indicating insensitivity to the analyte used.

A modified version of the ZDPlasKin package^34^ is used to integrate the above ODEs. The 0D system follows an ideal gas fluid parcel under atmospheric pressure, where the ideal gas law is enforced by adjusting the total number density according to the temperature profile. The initial condition corresponds to a location inside the analyte flow channel upstream of the ICP coil, where the flow is at room temperature and the analyte molecules are not dissociated. The initial species concentrations are calculated using the experimental molecular flow rates ${\dot{N}}$ given in Table 1 as:

6 $$\begin{aligned} n_{0,i}=\frac{P_0}{k_BT_0}\frac{{\dot{N}}_i}{\sum _j{\dot{N}}_j} \end{aligned}$$

where $n_{0,i}$ is the initial number density of molecule *i*, $P_0=1$ atm, and $T_0=300$ K. An analyte channel Ar flow rate of 1 L/min is used for this calculation. We assume that the representative Lagrangian streamline experiences limited mixing with Ar flow from the outer channel, such that the Ar to analyte mixing ratio remains constant throughout the simulation.

### Fit assessment

Lastly, the modeled chemical evolution is compared with the experimentally observed evolution to assess the agreement of the modified reaction mechanism. This comparison requires converting number density outputs from the evaluation step to corresponding emission intensity signals. Here, we employ the simplifying assumption that the PFR plasma is optically thin and self-absorption effects can be neglected. The synthetic intensity (in units of power per volume) due to an electronic de-excitation from an upper state 2 to a lower state 1 is calculated as:

7 $$\begin{aligned} I = \frac{hc}{4\pi \lambda }n_{2}A_{21} \end{aligned}$$

where *h* is the Planck constant, *c* is the speed of light, $\lambda$ is the line wavelength, $n_{2}$ is the excited state population, and $A_{21}$ is the transition probability. For an atomic transition, the number density of atoms for electronic level *e* is expressed in terms of the total species number density *n* as^35^:

8 $$\begin{aligned} n_{e} = n\frac{g_{el,e}}{q_{el}}\exp \left( -\frac{\Delta E_{e0}}{k_BT}\right) \end{aligned}$$

where $g_{el,e}$ and $\Delta E_{e0}$ are the statistical weight and the energy with respect to the ground state of level *e*, respectively, $k_B$ is the Boltzmann constant and $q_{el}$ is the electronic partition function:

9 $$\begin{aligned} q_{el} = \sum _e g_{el,e} \exp \left( -\frac{\varepsilon _{el,e}}{k_BT}\right) \end{aligned}$$

which is a weighted sum of populations across all electronic states. The electronic partition function for the uranium atom is calculated using energy levels from atomic spectroscopy databases^36–38^. Uranium has a strong atomic emission line at 591.5 nm, that is used for experimental comparisons in this study.

For a hetero-nuclear diatomic molecular transition, the number density of particles at a given level is calculated as:

10 $$\begin{aligned} n_{e,J,v} = n\frac{g_{el,e}}{q_{int}}(2J+1)\exp \left( -\frac{\Delta E_{e0,J0,v0}}{k_BT}\right) \end{aligned}$$

where the excited level is described by the electronic, rotational, and vibrational quantum numbers *e*, *J*, and *v*, respectively. Here, an approximate expression is used for the total internal partition function $q_{int}$ across these states:

11 $$\begin{aligned} q_{int} = \sum _e g_{el,e} \exp \left( -\frac{\varepsilon _{el,e}}{k_BT}\right) q_{rot,rr}q_{vib,ho}q_{corr} \end{aligned}$$

where the rigid rotor and harmonic oscillator terms $q_{rot,rr}$ and $q_{vib,ho}$ are given by:

12 $$\begin{aligned} q_{rot,rr} = \frac{k_BT}{B_e} \qquad q_{vib,ho} = \frac{\exp (-\omega _e/2k_BT)}{1-\exp (-\omega _e/k_BT)} \end{aligned}$$

where $B_e$ is the rotational constant and $\omega _e$ is the harmonic vibrational frequency, both expressed in units of energy. The approximate first order correction term $q_{corr}$^35,39^ accounts for anharmonic non-rigid motion and rovibrational coupling as:

13 $$\begin{aligned} q_{corr} = 1 - \frac{2k_BTD_e}{B_e^2} + \frac{\alpha _e}{B_e}\left[ \exp \left( \frac{\omega _e}{k_BT}\right) -1\right] ^{-1} + \frac{2\omega _e\chi _e}{k_BT}\left[ \exp \left( \frac{\omega _e}{k_BT}\right) -1\right] ^{-2} \end{aligned}$$

where $D_e$ is the centrifugal distortion constant, $\alpha _e$ is the rotational-vibrational coupling constant, and $\chi _e$ is the anharmonicity constant. In the above expression, $D_e$, $\alpha _e$, and $\omega _e\chi _e$ are formulated in units of energy. Owing to the complexity of the crowded UO emission spectrum, only the ground state spectroscopic constants have been previously estimated in literature^40–44^. The spectroscopic constants and energy levels estimated by Konings et al.^44^ will be used here to estimate the emission intensity of the 593.55 nm UO band. This band is assumed to be dominated by the [16.845]5–X(1)4 transition (a 0–0 transition) observed by Kaledin et al.^43^. Although many closely spaced rovibrational lines^14^ also contribute to this band, they are not treated in the current work due to both a lack of spectroscopic constants and the limited resolution of the spectrometer used.

In theory, Eq. (7) could be used to relate measured emission intensities to absolute number densities using a well-known reference signal (i.e. strong Ar line). However, the transition probabilities of ${{\mathrm{UO_x}}}$ species have uncertainties as high as 50%^45^, which prohibits an accurate determination of emission-based number densities. To circumvent this limitation, we instead normalize both the experimental and modeled emission intensity profiles such that the objective function minimizes the difference in the shapes, rather than magnitudes, of the two profiles. The strongest (most upstream) emission signal is used as the normalization point such that the experimental and modeled emission curves are scaled to have the same magnitude at the strongest emission point. Unphysically low values of the synthetic U emission signals (i.e. too low to produce a detectable signal) are avoided by including a penalty term in the objective function that checks the maximum ratio of synthetic emission intensities $I_{U/UO}=I_U/I_{UO}$. As the name implies, the penalty term mathematically penalizes solutions that fall outside the desired range of $I_{U/UO}$ values and is given further below. Based on the above considerations, the following root mean square error (RMSE) objective function ($\phi$) is formulated:

14 $$\begin{aligned} \phi = \sqrt{\frac{1}{2C}\Bigg [(1-\phi _p)^2 + \sum ^C_c(1-R^2_{lin,c})^2 + \sum ^C_c(1-R^2_{log,c})^2\Bigg ]} \end{aligned}$$

where $\phi _p$ is the penalty function and $R^2_{lin,c}$ and $R^2_{log,c}$ are the weighted linear and logarithmic coefficients of determination for emission curve *c* given by:

15 $$\begin{aligned} R^2_{lin,c}&= 1-\frac{\sum _i^{\textrm{N}} w_i\left[ I^{exp}_{i} - I^{calc}_{i}({\varvec{k}})\right] ^2}{\sum _i^{\textrm{N}} w_i\left[ I^{exp}_{i} - {\bar{I}}_{lin}^{exp}\right] ^2} \end{aligned}$$

16 $$\begin{aligned} R^2_{log,c}&= 1-\frac{\sum _i^{\textrm{N}} w_i\left[ \log (I^{exp}_{i}) - \log (I^{calc}_{i}({\varvec{k}}))\right] ^2}{\sum _i^{\textrm{N}} w_i\left[ \log (I^{exp}_{i}) - {\bar{I}}_{log}^{exp}\right] ^2} \end{aligned}$$

where $w_i=W_i/\sum _i^{\textrm{N}}W_i$ is a normalized statistical weight, ${\bar{I}}_{lin}^{exp}=\sum _i^{\textrm{N}}w_iI_i^{exp}$ and ${\bar{I}}_{log}^{exp}=\sum _i^{\textrm{N}}w_i\log (I_i^{exp})$ are the weighted linear and logarithmic means of measured normalized emission intensities, respectively, and $I^{exp}_i$ and $I^{calc}_i({\varvec{k}})$ are the measured and calculated normalized emission intensities at time *i*, respectively. *N* represents the number of experimental data points comprising emission curve *c*, and *C* represents the total number of emission curves used. The use of both linear and logarithmic coefficients here is intended to ensure that both large amplitude changes (which dominate the linear fit) and small amplitude changes (which dominate the log fit) in the emission signals are well fitted. The former is constrained by the rapid emission drop-off near the RF coil and the latter by the more gradual emission decay further downstream. The statistical weight $W_i$ of each data point is given by:

17 $$\begin{aligned} W_i = 1-\left( \sqrt{\frac{\sum _j^B\left[ S^{bck}_j-{\bar{S}}^{bck}\right] ^2/B}{\sum _l^M\left[ S^{mol}_l-{\bar{S}}^{bck}\right] ^2/M}}\right) _i \end{aligned}$$

where $S_j^{bck}$ and $S_l^{mol}$ are the background and molecular emission signals consisting of *B* and *M* experimental data points, respectively. This weighting quantifies the strength of a given emission line relative to the strength of the background. Therefore, weakly emitting lines on the order of the background will be weighted lighter when evaluating the objective function compared to strong emission lines.

Due to the inverse correlation between $I_{U/UO}$ and $n_{UO}/n_U$, the following penalty term is used:

18 $$\begin{aligned} \phi _p = 1 - \frac{\log (I_{U/UO}/{\bar{I}}_{U/UO})^2}{\log (I^{min}_{U/UO}/{\bar{I}}_{U/UO})^2} \end{aligned}$$

where ${\bar{I}}_{U/UO}=11$ and $I^{min}_{U/UO}=1$. The function most strongly discourages solutions where $n_{UO}/n_U \gg 10$ to prevent locating solutions with good fitness but unreasonably low U densities. The transition probability values used to calculate $I_U$ and $I_{UO}$ are $A_{U,21}=3.15 \times 10^6$ s$^{-1}$ for the 591.54 nm U line^46^ and $A_{UO,21}=3.8 \times 10^9$ s$^{-1}$ for the 0–0 head of the 593.55 nm UO band^47^, respectively. A worst-case scenario uncertainty of $\pm 50\%$^45^ is assumed for both of these values. From experimental maximum $I_{U/UO}$ values between 3 and 7, a rough range of $1< I_{U/UO} < 21$ for the modeled ratio is obtained. Calculating the species number densities corresponding to the lower bound $I_{U/UO}=1$, we find $n_{UO}/n_U\approx 120$. Accordingly, the mean and max values of $I_{U/UO}=11$ and $I_{U/UO}=21$ yield $n_{UO}/n_U\approx 11$ and $n_{UO}/n_U\approx 5.5$, respectively.

The final part of the Monte Carlo loop consists of deciding whether to retain or discard the candidate reaction mechanism based on the fitness of the solution. This step builds up the set of reaction mechanisms that will be passed to the genetic algorithm for further optimization. The criteria for keeping candidate solutions can be adjusted as needed, but are typically based on exceeding a threshold value for one or more of the fitness metrics calculated above. For example, a simple criterion such as $R^2_{lin}>0.5$ for all solution curves can be used. If a solution is retained, then the statistical values calculated as part of Eq. (14) and the corresponding sets of modified rate parameters from Eq. (3) are stored. It is preferable to have a lax selection criterion at this stage in order to build up a sizable number of candidate mechanisms. The mechanisms passed to the genetic algorithm are then selected from these candidates based on the fitness metric used by the algorithm.

### Genetic algorithm details

**Figure 4:**
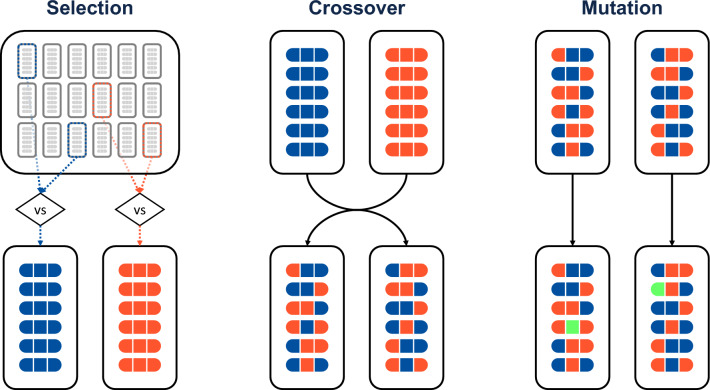
Diagram illustrating the tournament selection, uniform crossover, and mutation operations of the genetic algorithm portion of the Monte Carlo Genetic Algorithm (MCGA). In this example, each “individual” or “chromosome” (reaction mechanism) is composed of 18 “genes” (6 reaction channels with 3 rate coefficients each).

The genetic algorithm (GA) implementation used here employs a mix of operations and ideas from several previously published approaches^6,48,49^. As is often the case, the choice of GA parameters is largely heuristic based on the observed rates of convergence, fitness improvement, and solution variability. Therefore, while the GA implementation employed herein is not necessarily optimal, it nevertheless performs sufficiently well to reliably improve population fitness while maintaining a baseline level of diversity.

In the GA, the population of each new generation is created by performing several genetic operations on the current population, as illustrated in Fig. 4. Here, a population refers to a set of individuals, each of which is a reaction mechanism with rate coefficients modified via Eq. (3). Each mechanism contains a number of parameters equal to the number of reactions times the number of Arrhenius coefficients (i.e. 3 parameters per reaction). The new generation begins by directly transferring a select number of the fittest mechanisms (elites) from the current generation to the new one. Here, we select a number of elites equal to 5% of the total population. Next, mate selection is performed using a *k*-tournament of size $k=2$ with a 80% probability of selecting the fittest mechanism in the tournament. That is, each tournament randomly picks 2 mechanisms from the current population, and the fittest of the two is selected with 80% probability (otherwise the other mechanism is selected). The entire population participates in the selection process, including elites. Each pair of distinct mates picked in this way then has a chance to reproduce and/or mutate before being added to the new population. The mates may be converted into offspring through uniform crossover, wherein each parameter (rate coefficient) is switched between the two mates with equal probability, resulting in two offspring mechanisms. In this work, the probability of crossover reproduction for a given mate pair is 65%. Regardless of whether reproduction takes place, the two mechanisms may then also undergo mutation. In this operation, each parameter (rate coefficient) of a mechanism is randomized within previously specified bounds with 0.8% probability. The probability of mutation is kept low since even a single re-randomization operation may produce a drastic change in the mechanism behavior. Since both the reproduction and mutation operations may or may not occur for a given mate pair, it is possible for a given pair to simply pass on to the next generation (i.e. survive). This does not remove them from the selection pool, so mate pairs can both survive and reproduce/mutate. However, each mechanism in the new population must be unique, so duplicate mechanisms are checked and discarded as needed. The above operations of selection, reproduction, and mutation are repeated until a new population of the same size as the original is generated.

Since the fitness of the elites is already known, only 95% of each new generation must be evaluated. There are various methods for parallelizing this process^50^, some of which involve creating sub-populations either to allow for non-synchronized evaluation or to allow for additional evolutionary operations (i.e. migration). Here, we use a simple controller-worker parallelization wherein only the task of evaluation (which is the most computationally demanding task) is split between the number of available processors. Note that, for added genetic diversity, the first generation is constructed by supplementing the initial population (the fittest Monte Carlo generated mechanisms) with an equal number of mechanisms with randomized rate coefficients (within allowed bounds)^6^.

## Results

### Experimental emission spectra datasets

**Figure 5:**
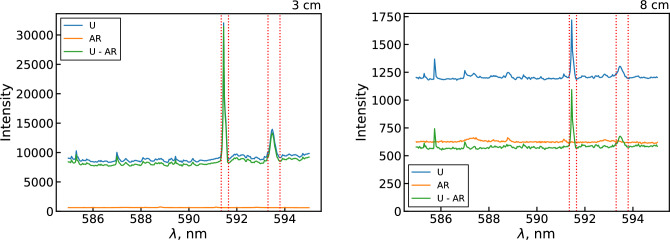
Plots showing the uranium, background (argon), and background subtracted uranium emission spectra measured at 3 and 8 cm away from the RF coil. The vertical lines denote the locations of the 591.5 nm U line and the 593.55 nm UO band.

Several uranium optical emission datasets were collected from the PFR to inform the optimization. Parameters varied between datasets included the regions of observation, the flow temperature (varied via flow rates and RF power), and the oxygen concentration in the analyte flow, as summarized in Table 4. Examples of uranium and background emission spectra collected 3 and 8 cm away from the RF coil are shown in Fig. 5. The background (labeled “AR”) spectra were measured with only argon and nebulized water flowing through the analyte channel. Therefore, the measured background includes emission due to de-excitation of background species, continuum (thermal) radiation, inherent instrument noise, and any other stray background light. The uranium spectra show significant background signals even when corrected for the measured background, as observed in previous uranium spectroscopy studies^1^. This ‘excess’ background likely occurs due to the multitude of closely spaced uranium emission lines in the visible spectrum combined with the limited resolution of typical spectrometers. As such, extracting the emission intensity of a given uranium line or band requires first subtracting out this additional background signal. Doing so accurately would either require using a much higher resolution spectrometer or attempting to deconvolve the peaks of interest using a complete uranium oxide spectral model, both of which lie outside the scope of this work. Instead, we assume that the background peaks are both much weaker and more numerous than the bands of interest, such that a simple offset can be used to approximately separate one from the other. It is difficult to assess the uncertainty introduced by this assumption without a full spectral model of uranium oxide emission. Qualitatively, it should have a greater impact on the 593.55 nm UO band than the atomic 591.5 nm U line, as the former signal is typically weaker than the latter. Furthermore, the UO band consists of several closely spaced rovibrational lines that require a much higher spectral resolution (order of 0.004 nm^14^) to properly resolve. Lastly, the UO partition function calculation is approximate due to the limited information on the internal states of the system^44^. Therefore, likely the greatest uncertainty in the current optimization procedure lies with measuring and calculating the signal due to the 0–0 head of the 593.55 nm UO band.

**Table 4:** Summary of experimental parameters used for each dataset.

	Position range (cm)	Position increment (cm)	Outer Ar flow rate (L/min)	RF power (W)	Added $\hbox {O}_2$ flow rate (mL/min)	Number of acquisitions (–)	Exposure time (s)
Dataset 1	3–8	0.1	12	784	0, 10, 20	1	10
Dataset 2	3–8	0.1	10	840	0	1	10
Dataset 3	1–2.5	0.1	10	840	0	10	1

The measured argon and water background is negligible compared to the uranium background for most locations, such that correcting for it has no noticeable impact on the results. Even for downstream locations where the argon and water background was on the same order of magnitude as the uranium background, the contribution of the argon and water background in the spectral regions of interest was minimal. Therefore, background measurements were not performed for subsequent datasets, and instead a constant offset based on the total uranium background was applied. This is accomplished by selecting a wavelength range (586 to 586.5 nm) as a reference background region (due to lack of visible peaks across all locations) and using the mean signal in this range as the offset value. After applying this offset, the intensities of the 591.5 nm U line and 593.55 nm UO band are calculated by integrating over the corresponding peaks.

**Figure 6:**
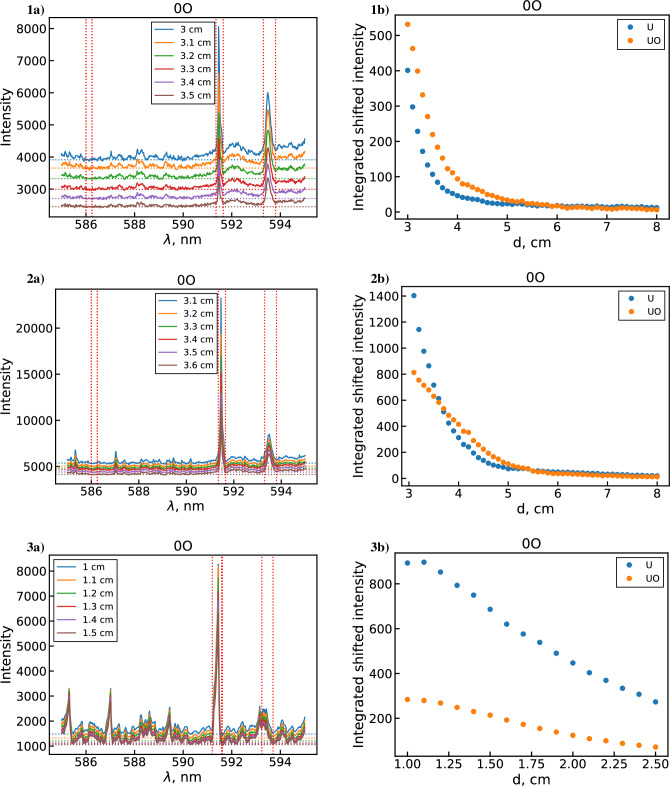
Example uranium spectra (**a**) and corresponding integrated background subtracted line intensities (**b**) for datasets 1, 2, 3 with no added $\hbox {O}_2$ flow.

**Figure 7:**
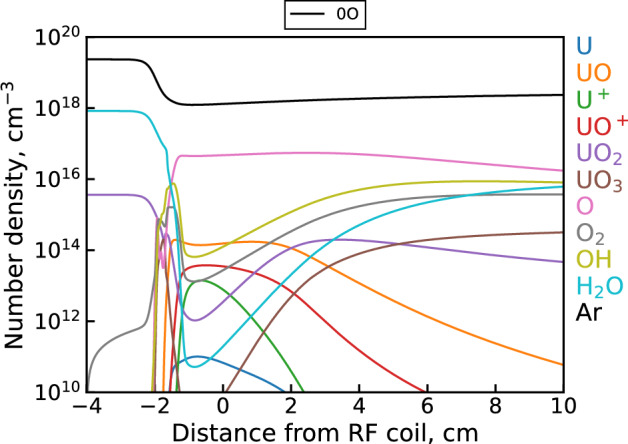
Unoptimized 0D ${{\mathrm{UO_x}}}$ model^5^ results showing select species profiles in the flow according to the temperature profile shown in Fig. 3.

Figure 6 shows examples of uranium spectra from each dataset along with the corresponding integrated background subtracted intensity values for U and UO over all measured locations. The integrated intensity plots show both the U and UO signal monotonically decreasing over the entire observation range. This holds true even when accounting for the decrease in emission intensity due to the temperature decline over this distance. Since the temperatures in the upstream (1 cm) region are expected to be high enough ($\sim$4500 to 5000 K) to at least partially dissociate UO, the UO concentration and emission intensity is expected to initially increase moving downstream. The observed UO trend, however, suggests that UO formation instead happens even further upstream of the first observation point here (i.e. in the coil region). The origin of this behavior can be explained by modeling the species profiles in the flow, as shown in Fig. 7. At first, the analyte flow consists purely of the constituent reactive molecules $\hbox {UO}_2$, $\hbox {H}_2$O, $\hbox {NO}_3$ (not pictured) and the Ar carrier gas at room temperature. Moving downstream, the flow encounters a sharp temperature gradient at $-2$ cm that decreases the gas number density (via the ideal gas law) and produces rapid dissociation, excitation, and ionization of the analyte molecules. Note that the uranium ionization in this case is almost entirely due to the ${\mathrm{U+O}}$ associative ionization channel of the unoptimized mechanism. While $\hbox {H}_2$O and $\hbox {NO}_3$ are effectively fully dissociated into their atomic components, the flow passes through the plasma region too quickly to fully dissociate uranium oxide molecules. As a result, we see two UO peaks appear; the first due to the initial temperature gradient breaking apart $\hbox {UO}_2$ in the analyte and the second due to the downstream cooling allowing ${\mathrm{U+O}}$ reactions and electron recombination of UO$^+$ to take place. After this point, gradual cooling induces formation of higher uranium oxides ($\hbox {UO}_2$ and $\hbox {UO}_3$) which eventually deplete the previously formed UO molecules. The monotonic decrease in UO signal observed in the above datasets corresponds to this last regime. Note also that due to the availability of free oxygen from the dissociation of $\hbox {H}_2$O and $\hbox {NO}_3$, uranium saturates toward a higher oxide ($\hbox {UO}_3$) than its initial analyte form ($\hbox {UO}_2$).

**Figure 8:**
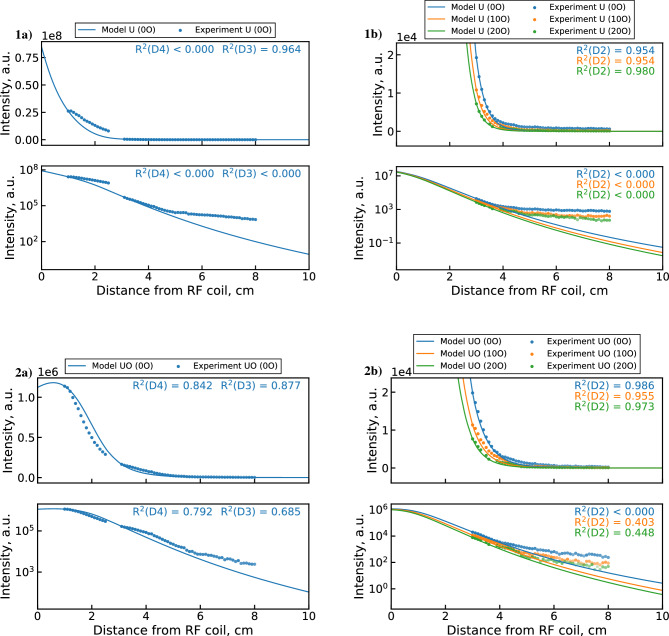
Synthetic emission profiles of (1) U and (2) UO (with linear and semi-log axes) generated by the unoptimized 0D ${{\mathrm{UO_x}}}$ model^5^ compared with (**a**) combined datasets 2 & 3 measurements and (**b**) dataset 1 measurements. The transparency of experimental points indicates signal-to-background ratio.

Comparisons of synthetic emission profiles and measured emission data are shown in Fig. 8. The synthetic UO emission profile (2a) shows surprisingly close agreement with the experimental data. However, the model slightly underpredicts the upstream UO depletion rates and overestimates the downstream rates. Poorer agreement is observed for the U profile (1a), which decreases faster in the model in both upstream and downstream regions. An interesting feature of the U emission data is observed in the semi-logarithmic plots. Namely, the U emission signal appears to saturate toward a minimal value after around 4–5 cm. The signal to background ratio for the 591.5 nm U line at these locations remains consistently high, so this behavior cannot be attributed to instrument noise or the uranium background. Indeed, this behavior is not observed for the UO band, which is generally weaker than the U line and approaches background after 6 cm. One possible explanation is that this downstream U signal originates from scattered light emitted in the upstream portion of the PFR. Since the 591.54 nm U emission line is more intense than the 593.55 nm UO band in the upstream region, the scattered light could disproportionately contribute to the downstream U line intensity. Given that this observation may be unrelated to chemical reactions, it merits special consideration during the optimization procedure, as discussed later.

### Monte Carlo: exploration of parameter space

As previously discussed, the purpose of the Monte Carlo step of the MCGA is to perform a preliminary survey of the problem parameter space and generate a starting population for the genetic algorithm. As such, a relaxed criterion for retaining candidate mechanisms is used, only requiring a positive linear-scale coefficient of determination for all modeled species profiles (i.e. $R^2_{lin}>0$). This criterion was satisfied by 8.61% of generated mechanisms from 2.3 million samples. These 200,000 candidate mechanisms could be used as a starting population for the GA portion of the algorithm. However, to keep the run time of the GA reasonable, only a subset of the candidate mechanisms is used. This is done by first sorting the mechanisms according to two versions of the objective function $\phi$ previously defined in Eq. (14). The two objective functions are: $\phi _1$, which includes all terms in the objective function:

19 $$\begin{aligned} \phi _1 = \sqrt{\frac{1}{N}\left[ (1-\phi _p)^2 + \sum ^{1,2,3}_D\sum ^{U,UO}_s(1-R^2_{lin,D,s})^2 + \sum ^{1,2,3}_D\sum ^{U,UO}_s(1-R^2_{log,D,s})^2\right] } \end{aligned}$$

and $\phi _2$, which excludes $R^2_{log}$ terms for atomic uranium emission:

20 $$\begin{aligned} \phi _2 = \sqrt{\frac{1}{N}\left[ (1-\phi _p)^2 + \sum ^{1,2,3}_D\sum ^{U,UO}_s(1-R^2_{lin,D,s})^2 + \sum ^{1,2,3}_D\sum ^{UO}_s(1-R^2_{log,D,s})^2\right] } \end{aligned}$$

where *D* refers to the datasets (including various oxygen flow conditions), *s* refers to the species, and *N* represents the total number of terms in each case. The latter formulation is included here due to the anomalous saturation of the downstream uranium emission signal seen in Fig. 8, which dominates the log space experimental U curve. If this effect is due to chemical processes, then we expect it to be well matched by the MCGA algorithm using $\phi _1$. However, if the saturation behavior is not capture when using $\phi _1$, then the effect may be non-chemical in nature, in which case it should not be used to constraint the reaction mechanism and $\phi _2$ should be used instead.

**Figure 9:**
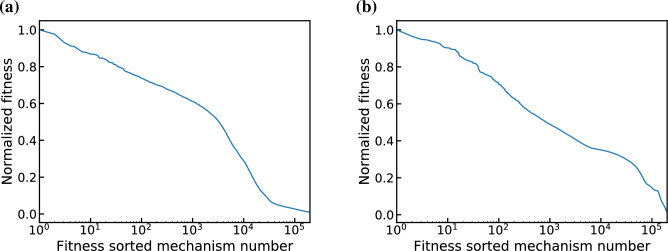
MC generated candidate mechanisms sorted by fitness values ($1/\phi$) evaluated using (**a**) $\phi _1$ (Eq. 19) and (**b**) $\phi _2$ (Eq. 20) objective functions, which differ only by the exclusion of U emission in $\phi _2$, and normalized with respect to the maximum fitness value for each objective function.

Figure 9 plots normalized fitness-sorted MC generated mechanisms using either $\phi _1$ or $\phi _2$ as the objective function. Fitness here is defined as $1/\phi$ due to our formulation of $\phi$ being minimized for the best fit. Note that both $\phi$ and fitness are statistical, rather than physical, quantities. Furthermore, the fitness values are only meaningful in the context of the objective function used to calculate them and cannot be compared across objective functions. For convenience, the fitness values here are normalized with respect to the maximum fitness value for the corresponding objective function. For both objective functions, only about 5% of the generated mechanism have fitness values within 67% of the maximum fitness. Furthermore, only a few hundred mechanisms (out of 200,000) fall within the top 20% of fitness. This subset of top mechanisms serves as the initial population for the genetic algorithm, as discussed below.

### Genetic algorithm: fitness optimization

Having chosen the target objective functions, the genetic algorithm can now be used to optimize the ${{\mathrm{UO_x}}}$ reaction mechanism for the experimental conditions used in this study. To test the reliability of the genetic algorithm, we perform four separate GA optimizations. These optimizations represent combinations of two input settings with two options each. The first input setting sets the objective function used, with the two options being $\phi _1$ and $\phi _2$ as described by Eqs. (19) and (20), respectively. The second input setting sets the initial population used (i.e. starting set of candidate reaction mechanisms), with the two options being the 200 best MC generated mechanisms according to either $\phi _1$ or $\phi _2$. Therefore, two optimizations use the $\phi _1$ and $\phi _2$ objective functions initialized with the corresponding 200 fittest MC mechanisms according to that objective function. We will refer to these as the optimal population runs. The other two optimizations instead swap the initial populations used for each objective function, such that the starting GA population is sub-optimal in each case. That is, the $\phi _1$ optimization is started with the fittest population according to $\phi _2$ and vice versa. This tests whether the GA can reliably obtain the same optimal fitness regardless of the starting population. The overlap between the fittest mechanisms for the two objective functions is only 2 mechanisms out of 200. Lastly, for both the optimal and sub-optimal populations, 200 MC samples of arbitrary fitness (i.e. generated with no constraints/selection criteria) are added to the initial population to provide additional diversity for the optimization^6^. Therefore, the total starting GA population for each run consists of 400 mechanisms (individuals).

**Figure 10:**
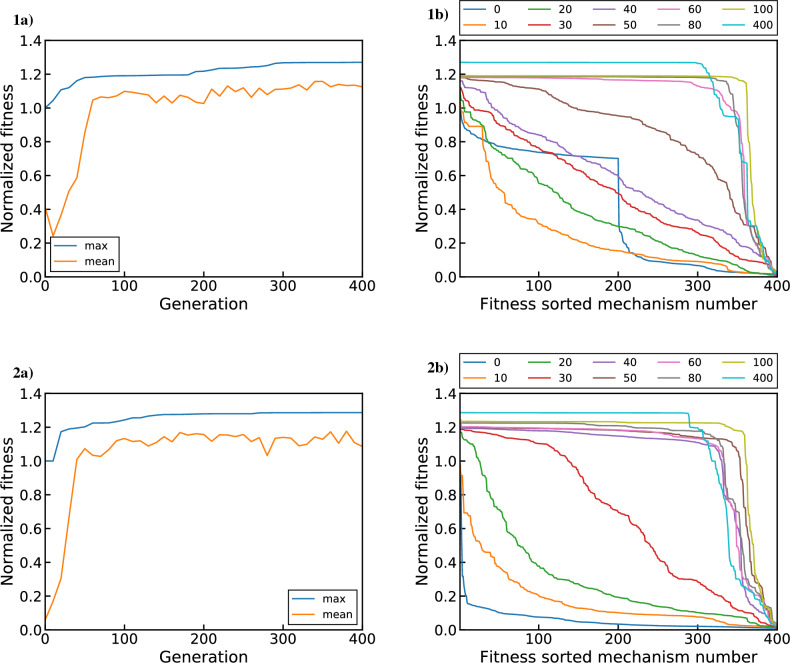
GA optimization results using $\phi _1$ (all $R^2_{log}$ terms) initialized with (1) optimal and (2) sub-optimal MC generated mechanisms. Plots (**a**) show the mean and maximum normalized fitness as a function of generation while plots (**b**) show sorted normalized fitness distributions for select generations.

Results from GA optimizations using $\phi _1$ (all $R^2_{log}$ terms) with optimal and sub-optimal initial populations are shown in Fig. 10. Subplots 1a and 1b illustrate results for the optimal case while subplots 2a and 2b show corresponding results for the suboptimal case. Note that the total number of generations plotted here (400) is not correlated with the number of mechanisms in the population (also 400). The optimal and sub-optimal starting populations are shown by the blue curves (labeled 0) in 1b and 2b, respectively. The optimal case (1b) clearly shows the fitness difference between the 200 best MC mechanisms and remaining 200 mechanisms of arbitrary fitness by the discontinuity in the middle. This difference is not evident in the sub-optimal case (2b), which shows the low overall fitness of the starting population aside from a few high fitness mechanisms. Looking at the evolution of the max and mean population fitness (1a and 2a), we see that that the fitness increases most drastically within the first 40–60 generations. During this time, the diversity of the initial population is leveraged by crossover reproduction to rapidly locate high fitness regions in the parameter space. This improves the overall fitness of the population (as evidenced by the mean curve) and locates fitter individuals to displace the starting “elites” (increasing the max fitness). This can similarly be seen in the population fitness distributions (1b and 2b), where the shift from a concave to a convex shape reflects the increase in mean fitness. After the first 100 generations, much of the population is homogenized toward a similar fitness value, as evidenced by the plateau shape of the population fitness distributions (1b and 2b). Past this point, mutations maintain a baseline level of genetic diversity, as indicated by the persistence of a lower fitness population subset that makes up about 10–25% of the total population. This also allows parameter exploration to continue, leading to gradual population fitness improvement (1a and 2a) over the remaining 300 generations. After 400 generations, both the optimal and sub-optimal starting populations saturate toward a similar fitness value. Note that while the fittest starting mechanism is shared by the optimal and sub-optimal populations, the final fittest mechanisms differ. Curiously, the sub-optimal starting case possesses a higher fitness after 400 generations than the optimal case, but this is likely a stochastic occurrence.

**Figure 11:**
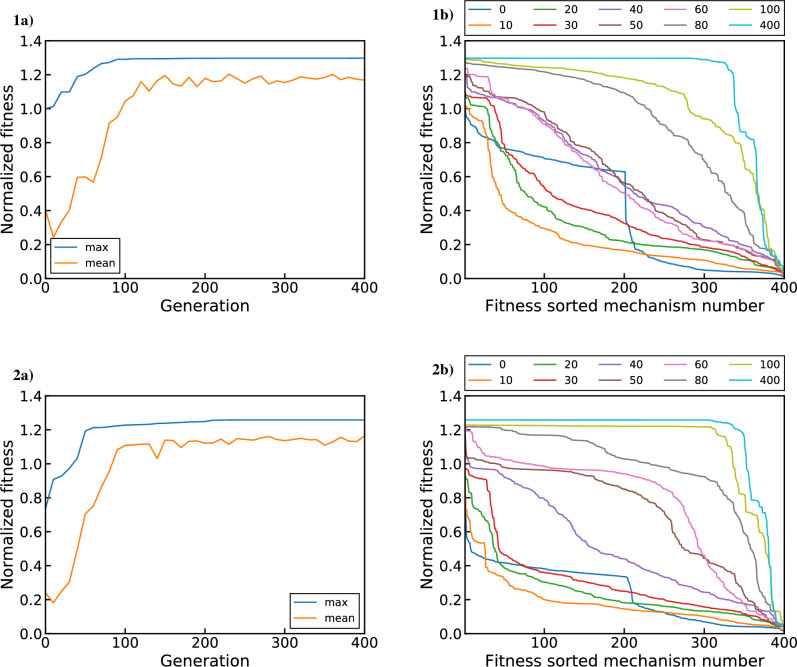
GA optimization results using $\phi _2$ (no $R^2_{log}$ for U) initialized with (1) optimal and (2) sub-optimal MC generated mechanisms. Plots (**a**) show the mean and maximum normalized fitness as a function of generation while plots (**b**) show sorted normalized fitness distributions for select generations.

Figure 11 presents the same set of plots for the GA optimizations using $\phi _2$ ($R^2_{log}$ excluded for U). The evolution of this GA population is very similar to that of the previously examined $\phi _1$ case. Despite the initially less fit population (both in mean and maximum), the sub-optimal run (2a and 2b) again arrives at a similar fitness as the optimal run (1a and 1b). In this case, the final fitness of the optimal case is higher than for the sub-optimal run, but this gap would likely close as the evolution is continued. Regardless, the above four runs show that the GA optimization performs reliably and produces similar fitness values regardless of the initial population used. However, the sub-optimal and optimal cases do not converge to an identical fitness value for either objective function over the 400 generations observed here. This may be due to the limited number of generations performed and the choice of GA properties dictating parameter exploration and convergence rates. This behavior may also be inherent to the optimization problem itself due to the limited range of conditions provided by the constraining data and the non-orthogonality of certain reaction channels. These considerations are illustrated by examining the rate coefficients predicted by the optimized populations, as discussed below.

**Figure 12:**
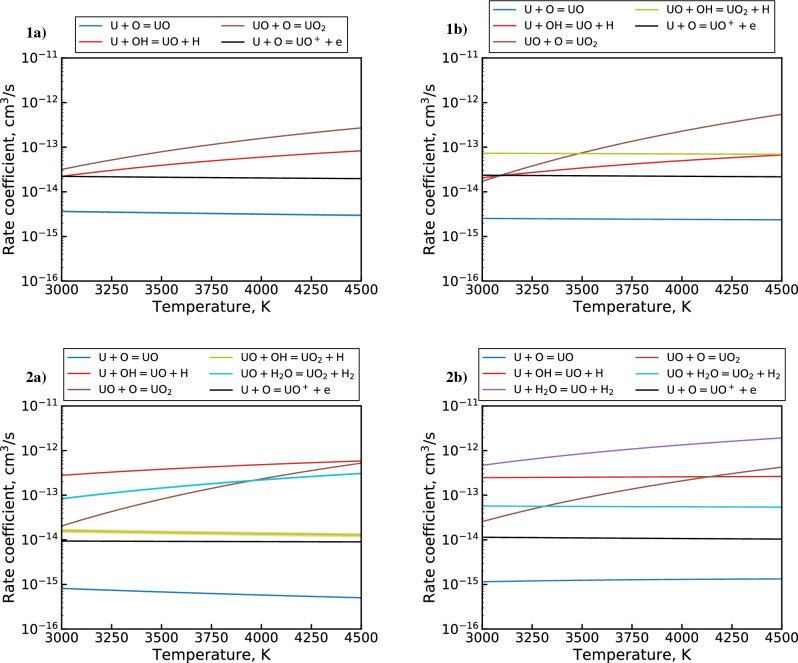
Mean reaction rate coefficients (${\bar{k}}$) of GA populations optimized using (1) $\phi _1$ and (2) $\phi _2$ initialized with (**a**) optimal and (**b**) sub-optimal MC generated mechanisms. The means are calculated from mechanisms falling within 0.1% of the top fitness ($\sim 300$ mechanisms in each case) for reactions that satisfy ${\bar{k}}>\sigma _k$ (where $\sigma _k$ is the standard deviation of *k*).

### MCGA output: dominant reaction channels

Figure 12 plots the mean rate coefficients of the main reaction channels from the four optimized populations. The dominant reactions are identified by examining the statistical variation in rate coefficients across the optimized populations. Only reactions for which the standard deviation of the rate coefficient does not exceed the mean are included (based on mechanisms within 0.1% of the top fitness). This condition is satisfied for at most seven out of the 12 optimized reactions channels, meaning that the remaining reactions are not well constrained by the current data. However, four reactions (R1, R4, R6, and R11 in Table 2) are consistently constrained, as indicated by their appearance in all optimization cases. The remaining three reactions (R5, R9, and R10) appear in only some of the optimized populations and are therefore only partially constrained. This is likely due to the non-orthogonality of the various reaction pathways. That is, channels like ${\mathrm{U + O}}$, ${\mathrm{U + OH}}$, and ${\mathrm{U + H_2O}}$ or ${\mathrm{UO + O}}$, ${\mathrm{UO + OH}}$, and ${\mathrm{UO + H_2O}}$ can compensate for one another since they perform the same operation on the constrained species (i.e. adding O to U or UO). Although the different oxygen flow conditions of dataset 1 constrain this behavior somewhat, the range of $\hbox {O}_2$ concentrations is limited and only downstream locations (>3 cm) are covered. These reactions could be better constrained in the future by performing upstream measurements over a wider range of $\hbox {O}_2$ conditions or with reduced $\hbox {H}_2$O concentrations (i.e. using a desolvating nebulizer). The current dataset is also limited in the range of temperatures and cooling rates covered, which also inhibits the location of a true global optimum. This is related to the temperature dependence of the Arrhenius rate expression, since similar reaction rates can be achieved using different combinations of coefficients if the reaction is active over a limited temperature range in the system. Based on the above observations, the lack of convergence toward a singular global optimum appears to stem more from the limited constraining data rather than from a shortcoming of the genetic algorithm. Even so, four reactions (R1, R4, R6, and R11 in Table 2) are consistently well constrained by the current optimization, demonstrating the reliability of the MCGA method and highlighting the dominant reaction channels for UO formation in the PFR.

**Figure 13:**
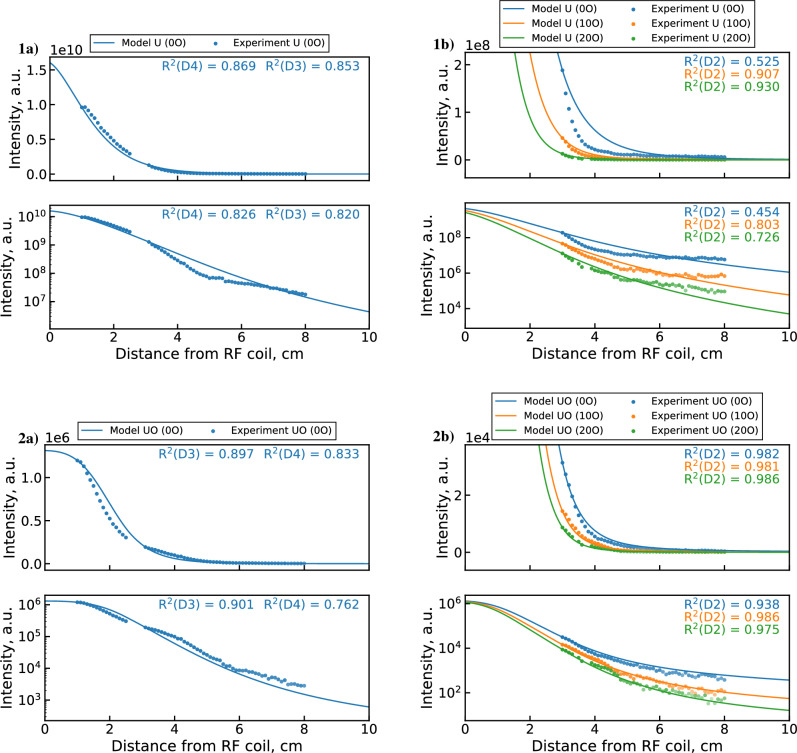
Synthetic emission profiles of (1) U and (2) UO (with linear and semi-log axes) generated by GA optimized mechanism using $\phi _1$ (all $R^2_{log}$ terms) compared with (**a**) combined datasets 2 & 3 measurements and (**b**) dataset 1 measurements.

Before performing a detailed analysis of the optimization results, we must decide which objective function to use by checking whether the saturation of the downstream U signal is captured by the $\phi _1$ optimized mechanism. Figure 13 shows the synthetic U and UO emission profiles produced by this mechanism compared to the full experimental dataset. Although the $\phi _1$ MCGA result produces improved fitting of the upstream data over the unoptimized mechanism (Fig. 8), the downstream (>3 cm) behavior of U is poorly captured. While the R$^2$ values appear to be adequate (outside of the 0 $\hbox {O}_2$ dataset 1 case), visual inspection reveals that neither the 3–5 cm decrease nor the 5–8 cm saturation behavior is well matched for any dataset. This suggests that the saturation may be driven by a non-chemical effect that is not accounted for in the current 0D treatment of the PFR. As discussed previously, this behavior is likely caused by optical scattering of the strong upstream U emission line. Therefore, we will consider it an invalid constraint for the current optimization problem and will focus our subsequent analysis on the $\phi _2$ optimized mechanism. As shown in the next section, the $\phi _2$ result captures the 3–5 cm decrease in U intensity while disregarding the subsequent signal saturation due to the exclusion of the log U term.

## Discussion

**Table 5:** Comparison between final MCGA optimized and previously constructed^5^ ${\mathrm{UO_x}}$ mechanisms.

No.	Reaction	$k^{{\textrm{a}}}_{mcga}$			$k_{lit}$		
		*A* (cm$^3$/s)	*n* (–)	$E_A/R$ (K)	*A* (cm$^3$/s)	*n* (–)	$E_A/R$ (K)
1	${\mathrm{U + O}} \rightleftharpoons {\textrm{UO}}$	$1.942 \times 10^{-11}$	− 1.25	209.88	–		
2	${\mathrm{U + O_2}} \rightleftharpoons {\mathrm{UO_2}}$	–			$3.360 \times 10^{-12}$	0.50	12910.0
3	${\mathrm{U + O_2}} \rightleftharpoons {\mathrm{UO + O}}$	–			$3.360 \times 10^{-12}$	0.50	5161.7
4	${\mathrm{U + OH}} \rightleftharpoons {\mathrm{UO + H}}$	$1.346 \times 10^{-13}$	0.32	5505.1	–		
5	${\mathrm{U + H_2O}} \rightleftharpoons {\mathrm{UO + H_2}}$	–			–		
6$^{{\textrm{b}}}$	${\mathrm{UO + O}} \rightleftharpoons {\mathrm{UO_2}}$	$1.950 \times 10^{-11}$	0.31	28020.3	$8.084 \times 10^{-13}$	0.27	3582.4
7$^{{\textrm{b}}}$	${\mathrm{UO + O_2}} \rightleftharpoons {\mathrm{UO_3}}$	–			$4.325 \times 10^{-11}$	− 0.23	− 7503.5
8	${\mathrm{UO + O_2}} \rightleftharpoons {\mathrm{UO_2 + O}}$	–			$3.800 \times 10^{-11}$	0.17	0.0
9	${\mathrm{UO + OH}} \rightleftharpoons {\mathrm{UO_2 + H}}$	$1.309 \times 10^{-12}$	− 0.56	2.03	–		
10	${\mathrm{UO + H_2O}} \rightleftharpoons {\mathrm{UO_2 + H_2}}$	$1.488 \times 10^{-13}$	0.36	10422.0	–		
11	${\mathrm{U + O}} \rightarrow {\mathrm{UO^+ + e^-}}$	$2.495 \times 10^{-14}$	− 0.12	51.3	$1.025 \times 10^{-12}$	0.50	0.0
12	${\mathrm{U + O_2}} \rightarrow {\mathrm{UO_2^+ + e^-}}$	–			$7.747 \times 10^{-14}$	0.50	0.0

^a^Only well-constrained reaction channels included here (see Fig. 12 for selection process).

^b^Literature rate calculated for reverse process, reversed here by fitting over 300 < T < 10,000 K.

**Figure 14:**
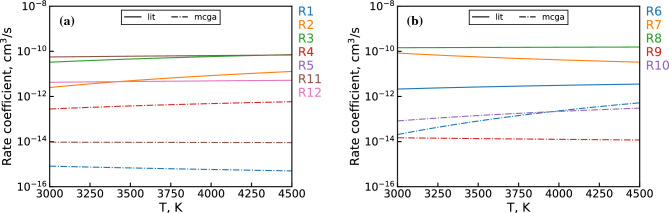
Comparison of literature^5^ (solid) and MCGA optimized (dash-dotted) rate coefficients for (**a**) ${\mathrm{U + H_xO_y}}$ and (**b**) ${\mathrm{UO + H_xO_y}}$ channels in Table 5. The rate coefficients are calculated over the given temperature range using the Arrhenius parameters from the table and Eq. (2).

The final set of optimized ${\mathrm{UO_x}}$ reaction channels and corresponding rate coefficients obtained by the MCGA are shown in Table 5. The table also includes relevant reaction channels from the previously constructed ${\mathrm{UO_x}}$ mechanism^5^. Only well-constrained rate coefficients are listed for the MCGA optimized mechanism (see Fig. 12). The rate coefficients for both mechanisms are plotted in Fig. 14 for the temperature interval $3000 \le T \le 4500$ K, which represents the range over which the optimization is performed (see Fig. 3). Since the unoptimized reaction mechanism did not consider interactions with ${\mathrm{H_xO_y}}$ molecules, only a few reaction channels are present in both mechanisms. This is exacerbated by the elimination of ${\mathrm{UO_x + O_2}}$ reaction channels by the MCGA optimization due to the relative abundance of OH in the PFR flow, as shown previously in Fig. 7. Therefore, only two reaction pathways (R6 and R11) can be directly compared between the two mechanisms. From Fig. 14, we can see that the MCGA optimized rate coefficient for the former reaction (R6) is at least an order of magnitude below the literature estimate. For the latter reaction (R11), the difference is even larger, around four orders of magnitude. In general, the unoptimized estimates lie an order of magnitude or more above the optimized rate coefficients. This is to be expected considering the literature estimates consist largely of barrierless first order hard sphere collision rate estimates, which are the theoretical upper limit of the reaction rates.

Table 5 and Fig. 14 provide additional insight into similarities and differences between the optimized and previously constructed reaction mechanisms. First, we note that the ${\mathrm{U+O}}$ pathway in the optimized mechanism is dominated by the associative ionization channel (R11) over the molecular association reaction (R1). This behavior is in partial agreement with a previous study^31^, which measured a much higher cross section for the associative ionization reaction (R11) compared to the molecular association reaction (R1). The study suggested that the molecular association channel is effectively closed due to the dominance of the associative ionization pathway. In the optimized mechanism, however, the two channels differ by only around an order of magnitude. Furthermore, the optimized R11 rate coefficient suggests a much lower cross section than observed in the aforementioned study. The optimized pathway is also found to be effectively barrierless, which is consistent with the study.

Next, we note some similarity in the optimized R4 and unoptimized R3 channels, particularly with regards to the activation energy^51^ (${\mathrm{E_A/R}}$ in Table 5). This may indicate that the abstraction mechanism for U colliding with either OH or $\hbox {O}_2$ proceeds in a similar manner. Comparing the two reactions further, we see that the optimized collision rate is about two orders of magnitude lower than the hard sphere estimate. Nevertheless, the overall optimized rate coefficient is still high relative to the other optimized reactions, as seen in Fig. 14.

Moving on to R6, we observe a large discrepancy in activation energy between the optimized result and our previous estimate. The activation energy in the unoptimized rate coefficient comes from an Eyring-estimate adjusted according to an analogous semi-empirical calculation for an Al oxidation mechanism^2,5^. The unoptimized barrier value is essentially a byproduct of the above adjustment, as the Eyring estimate itself is barrierless. The strong temperature dependence of this channel for all MCGA results (see Fig. 12) suggests that the sizable activation barrier of the optimized rate is physically significant. The collision rate for this channel is observed to be close to the hard sphere limit, which offsets the effect of the large activation barrier. Thus, the overall rate coefficient is within an order of magnitude of the unoptimized channel at 4500 K and is around the same value as R4 at this temperature, as Fig. 14 shows.

Next, the R9 abstraction reaction is found to be effectively barrierless and has an overall low collision rate comparable to the R11 associative ionization channel. The lack of an activation barrier for the analogous unoptimized R8 abstraction channel is simply due to a lack of literature information, so a definitive comparison between R8 and R9 is not attempted here.

Lastly, the remaining R10 reaction channel is found to have a collision rate similar to R4, albeit with a higher activation energy. Despite the relatively large rate coefficient, this reaction pathway is expected to be important only in the downstream portion of the PFR due to its dependence on $\hbox {H}_2$O, which reaches concentrations comparable to OH only at $> 7$ cm where temperature drops below 3000 K.

**Figure 15:**
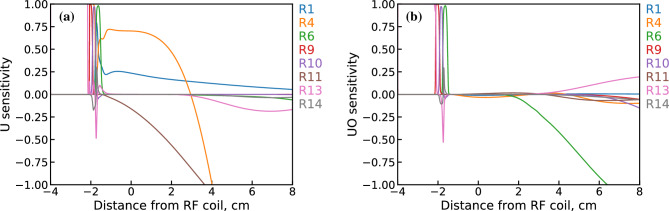
First-order sensitivity coefficients for (**a**) U and (**b**) UO using the MCGA optimized mechanism for dataset 2 & 3 conditions.

To analyze where the dominant reaction channels are most active in the PFR flow, we calculated the first-order sensitivity coefficients for the chemical kinetic system of equations^52^. The results for U and UO for dataset 2 and 3 conditions are shown in Fig. 15. In addition to the optimized channels, these plots include the fixed ${\mathrm{UO_2/UO_3}}$ formation reactions R13 and R14 from Table 3. From these plots we see that R1, R4, and R11 play the greatest role in the upstream (<3 cm) evolution of U, while R1 and R13 become dominant further downstream (>3 cm). The UO sensitivity plots show that the upstream UO evolution is relatively insensitive to the reaction mechanism. Further downstream, UO is most sensitive to R6 and R13. The remaining optimized channels from Table 5, R9 and R10, appear to make finer adjustments to the downstream UO evolution. Overall, the upstream behavior of the mechanism is constrained mainly by U data, while both U and UO measurements play a role in constraining the downstream behavior.

**Figure 16:**
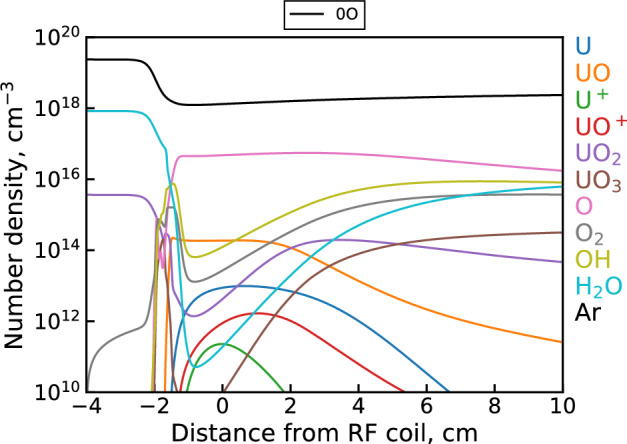
Modeled number density profiles of select species predicted by the MCGA optimized ${{\mathrm{UO_x}}}$ mechanism.

The species number densities produced by the optimized reaction mechanism are shown in Fig. 16. Compared to the unoptimized results of Fig. 7, the MCGA mechanism produces markedly less uranium ions in the coil region due to the lower R11 associative ionization rate. Although this results in a higher population of neutral U, it is still about an order of magnitude less abundant than UO. A peak upstream intensity ratio of $I_{U/UO}\approx 6$ is obtained using the optimized mechanism, which corresponds to $n_{UO}/n_U\approx 20$. A similar value is obtained across all the other MCGA optimizations. While this falls within the range of $I_{U/UO}$ values allowed by the penalty term, a thorough calibration of the absolute densities would be required to validate this result.

**Figure 17:**
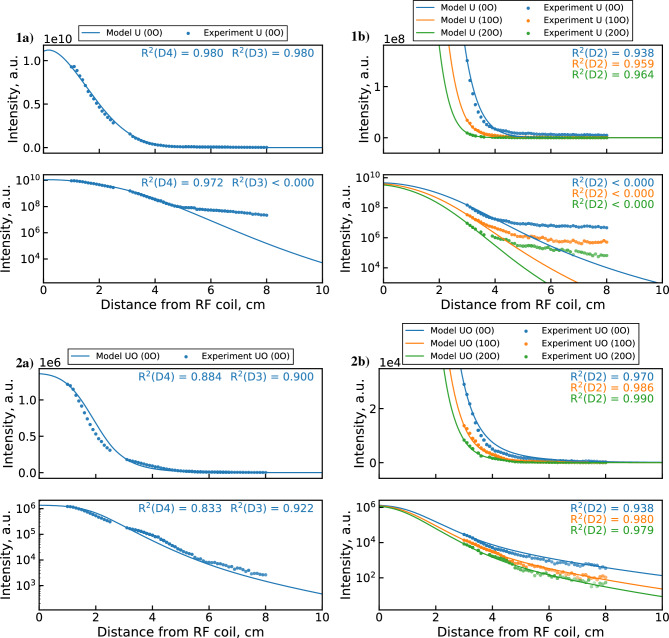
Synthetic emission profiles of (1) U and (2) UO (with linear and semi-log axes) generated by the GA optimized ${{\mathrm{UO_x}}}$ mechanism compared with (**a**) combined datasets 2 & 3 measurements and (**b**) dataset 1 measurements.

Lastly, to examine the fitness of the optimized mechanism, the final uranium synthetic emission profiles and constraining datasets are plotted in Fig. 17. As expected, the MCGA mechanism produces improved fitting of most experimental data when compared to the unoptimized results of Fig. 8. The largest discrepancy between the data and model is the downstream low amplitude saturation of the U emission signal, as seen in the semi-log plots. This arises from using the $\phi _2$ objective function, which excludes the saturation behavior as a constraint. However, the optimized mechanism produces an excellent fit of the upstream ($<5$ cm) U data. The mechanism also produces good fitting of both the upstream and downstream UO data. As discussed previously, the partial fitting of the upstream UO data provided by dataset 3 is limited by the approximate representative temperature profile used here.

## Conclusion

In this work, we investigated an MCGA-based approach for calibrating a ${{\mathrm{UO_x}}}$ reaction mechanism using emission measurements from PFR experiments. The selection of target reaction channels and their potential rate coefficients was made using a limited set of *a priori* assumptions. Consistency between the 0D PFR model and experiments was attained using a representative temperature profile in agreement with available temperature measurements. The Monte Carlo sampling and genetic algorithm steps were used to explore and optimize the problem parameter space, respectively, enabling refinement toward a fitness maximum. The resulting optimized ${{\mathrm{UO_x}}}$ reaction mechanism was analyzed, highlighting four dominant reaction channels that were consistently constrained across all attempted optimizations and three additional channels that were at least partially constrained. Notable among these channels is the involvement of the OH radical, which was not previously considered in the unoptimized mechanism. The optimized mechanism predicted slower kinetics for U and UO formation (with U kinetics being impacted more than UO) compared to the unoptimized mechanism, yielding rates that were 1–2 orders of magnitude or more below the prior hard sphere estimates. A notable feature of the optimized mechanism is the lower branching ratio between the associative ionization and neutral pathways of the ${\mathrm{U + O}}$ channel, which suggests the neutral pathway is not eliminated in favor of associative ionization as indicated in a previous study^31^.

Overall, this study demonstrates the viability of using a MCGA approach to optimize the chemical kinetic rate coefficients for a 0D PFR model using optical emission measurements. However, due to various limitations of the current study, we strongly caution against using the resulting optimized rate coefficients as is until additional optimization and validation is performed. A well validated reaction mechanism could be attained via the MCGA method after incorporating various refinements in a future work, as outlined below.

First, the importance of OH molecules for oxidizing uranium in the PFR suggests that both oxygen and hydrogen fugacity should be varied in future studies. This may be achieved by using a desolvating nebulizer, which removes most of the water from the analyte solution prior to introduction into the plasma. Second, additional temperature measurements in the coil region of the PFR would improve the consistency between model and experiments, which is important due to the sensitivity of the simulated chemical evolution to the temperature history. The consistency can be further refined by also considering the effects of mixing and radial diffusion downstream of the flow inlets. Third, the 593.55 nm UO band could be better resolved using a higher spectrometer grating. This would help eliminate some uncertainty in the calibration introduced due to the treatment of the UO band as the 0–0 head of the band. Fourth, the experimental dataset could be expanded to include information on higher uranium oxide formation (i.e. ${\mathrm{UO_2}}$ and ${\mathrm{UO_3}}$), using Fourier transform infrared spectroscopy (FTIR), for example. However, this would necessitate extending the atomic and diatomic emission calculations used here to larger molecules, which is potentially challenging for uranium species. Fifth, the modeling could be refined by performing detailed radiation transport calculations to quantify the impact of self-absorption and scattering on the simulated emission intensity throughout the PFR. Lastly, a relatively straightforward way to improve the MCGA generated mechanism is to use a larger dataset for the optimization. This includes measuring emission in both the upstream and downstream regions over a wider range of flow rates, temperatures, and analyte concentrations.

## Data Availability

The datasets generated during and/or analysed during the current study are available from the corresponding author on reasonable request.
